# Preferences for aspects of antenatal and newborn screening: a systematic review

**DOI:** 10.1186/s12884-019-2278-7

**Published:** 2019-04-16

**Authors:** Caroline M. Vass, Susanne Georgsson, Fiona Ulph, Katherine Payne

**Affiliations:** 1 Manchester Centre for Health Economics, The University of Manchester, Oxford Road, Manchester, M13 9PL UK; 2 The Swedish Red Cross University College, Stockholm, Sweden; 3 Division of Psychology & Mental Health, The University of Manchester, Oxford Road, Manchester, M13 9PL UK

**Keywords:** Newborn, Antenatal, Screening, Systematic review, Discrete choice experiment, Best-worst scaling, Preferences

## Abstract

**Background:**

Many countries offer screening programmes to unborn and newborn babies (antenatal and newborn screening) to identify those at risk of certain conditions to aid earlier diagnosis and treatment. Technological advances have stimulated the development of screening programmes to include more conditions, subsequently changing the information required and potential benefit-risk trade-offs driving participation. Quantifying preferences for screening programmes can provide programme commissioners with data to understand potential demand, the drivers of this demand, information provision required to support the programmes and the extent to which preferences differ in a population. This study aimed to identify published studies eliciting preferences for antenatal and newborn screening programmes and provide an overview of key methods and findings.

**Methods:**

A systematic search of electronic databases for key terms identified eligible studies (discrete choice experiments (DCEs) or best-worst scaling (BWS) studies related to antenatal/newborn testing/screening published between 1990 and October 2018). Data were systematically extracted, tabulated and summarised in a narrative review.

**Results:**

A total of 19 studies using a DCE or BWS to elicit preferences for antenatal (*n* = 15; 79%) and newborn screening (*n* = 4; 21%) programmes were identified. Most of the studies were conducted in Europe (*n* = 12; 63%) but there were some examples from North America (*n* = 2; 11%) and Australia (*n* = 2; 11%). Attributes most commonly included were accuracy of screening (*n* = 15; 79%) and when screening occurred (*n* = 13; 68%). Other commonly occurring attributes included information content (*n* = 11; 58%) and risk of miscarriage (*n* = 10; 53%). Pregnant women (*n* = 11; 58%) and healthcare professionals (*n* = 11; 58%) were the most common study samples. Ten studies (53%) compared preferences across different respondents. Two studies (11%) made comparisons between countries. The most popular analytical model was a standard conditional logit model (*n* = 11; 58%) and one study investigated preference heterogeneity with latent class analysis.

**Conclusion:**

There is an existing literature identifying stated preferences for antenatal and newborn screening but the incorporation of more sophisticated design and analytical methods to investigate preference heterogeneity could extend the relevance of the findings to inform commissioning of new screening programmes.

## Background

In many countries there are population-wide screening programmes for unborn (antenatal) or newborn babies [1]. These screening programmes are used to detect whether the baby is at a higher risk of certain conditions and therefore lead to an earlier diagnosis and inform timely decisions about their care and/or treatment [2]. The extent and content of target conditions for antenatal screening varies between, and sometimes within, countries. For example screening for sickle cell disease, thalassaemia and blood tests for infectious diseases may or may not be included. The probability of Down’s syndrome, Edwards’ syndrome and Patau’s syndrome can also be determined through ultrasound scans combined with blood tests [3], with high risk women being offered an invasive diagnostic test (such as amniocentesis or chorionic villus sampling) which raises the risk of a miscarriage but provides a conclusive result [4, 5]. New non-invasive prenatal testing (NIPT) is a maternal blood test that relies on the detection of fetal cell free DNA that can be used for analysis [6], is also becoming available in some countries, although often only privately [7]. Newborn screening often includes a physical and hearing examination, and newborn bloodspot screening. Newborn bloodspot screening programmes (NBSPs) are recognised as being one of the most significant public health achievements in the developed world [8, 9].

There have been changes and proposed future changes to the configuration of both antenatal and newborn screening programmes in Europe and around the world. Many countries have expanded their programmes to include more conditions in recent years [8, 10]. The change has been driven by the development of new technologies such as mass spectrometry in NBSPs [11] and DNA sequencing in NIPT [12]. However, expanded screening and new technology for testing means the options available are more complex than ever and require difficult trade-offs for the parent(s) who must balance the benefits and risks of participation [13, 14]. There is also a need for parents to be provided with the relevant information about the methods, associated risks, and the available options if the test results are positive [15]. Furthermore, some tests can give an indicative but inconclusive result leaving the parents uncertain or facing further risky tests for a definitive result to confirm the presence of an abnormality [16].

Various studies have sought to understand people’s (mothers’, fathers’ and healthcare professionals’) views for newborn and antenatal screening using qualitative research methods [17–20]. For example, one interview study of parents and professionals found they were concerned about information overload, insufficient information, how the information was provided and having autonomy in decision making [19]. Although able to illuminate factors of importance, qualitative studies are limited in their ability to determine the relative importance of different aspects of screening, the extent of heterogeneity within the population’s views or how changes to the screening programme may affect uptake.

A robust method for quantifying preferences, and the variation within, is a discrete choice experiment (DCE), and a recent 2002 [21] extension called best-worst scaling experiments (BWS), that can be embedded in a survey. In a DCE, respondents stated their preferred and/or least preferred option from a set of alternative goods or services described by the same attributes but differing in their amount (levels) [22]. From the choices made over a series of sets, it is possible to determine how the respondent balanced the different attributes and determine the relative impact each had on the probability of an option being selected. The data can also be analysed to understand how individuals balance the benefits and risks of participating in a healthcare programme and indirectly estimate the value they place on the attributes or the good or service as a whole.

Reviews of healthcare DCEs have found continued growth in studies using the method [23]. There has also been a rising interest in using the method to inform policy and regulatory decisions in the United States (US) and Europe [24–27]. There have been systematic reviews of the DCE literature for cancer screening [28] and women’s preferences for birth place [29]. However, no reviews have summarised studies that have elicited preferences for newborn or antenatal screening programmes. This study aimed to identify published studies that have elicited preferences for antenatal and newborn screening programmes and provide an overview of key methods and findings.

## Methods

A systematic review was conducted using a pre-defined protocol (available from the authors on request) based on standardised review methods [30]. For the purpose of this review, antenatal screening was defined as “population screening to identify people with a genetic risk, or a risk of having a child with a congenital or genetic disorder” through biochemical, genetic or ultrasound screening [31]. Newborn screening was defined as population screening of newborns to identify those at risk of congenital disorders to allow early intervention [32]. These definitions excluded childhood vaccination programmes.

### Search strategy

Medline, Embase, PsychInfo, Econlit and Maternity & Infant Care Database (MIDIRS) databases were searched on 10th October 2018 for key terms related to choice experiments including ‘discrete choice’, ‘choice experiment’, ‘stated preference’ and ‘best worst scaling’. These were combined with terms relating to antenatal and newborn screening including ‘foetal’, ‘fetal’, ‘foetus’, ‘fetus’, ‘prenatal diagnosis’, ‘prenatal testing’, ‘antenatal diagnosis’, ‘antenatal testing’, ‘antenatal screening’, ‘nipt’, ‘downs syndrome’, ‘trisomy’, ‘rapid aneuploidy’, ‘trisomies’, ‘karyotype’, ‘chromosom* abnormal*’, ‘cystic fibrosis’, ‘newborn screening’, ‘neonatal screening’, ‘newborn bloodspot’ and ‘bloodspot screening’.

Studies were excluded if they were written in a language other than English, did not report empirical choice data (for example, guidelines or other reviews), or were not related to antenatal or newborn screening. Studies that used rating or ranking conjoint experiments or an adaptive experimental design were also excluded. All abstracts were double screened.

### Data extraction and synthesis

Included studies were appraised using a checklist [22] specifically designed for DCEs. Extracted data were then tabulated and summarised as part of a narrative synthesis drawing upon the key findings of each article.

## Results

**Fig. 1:**
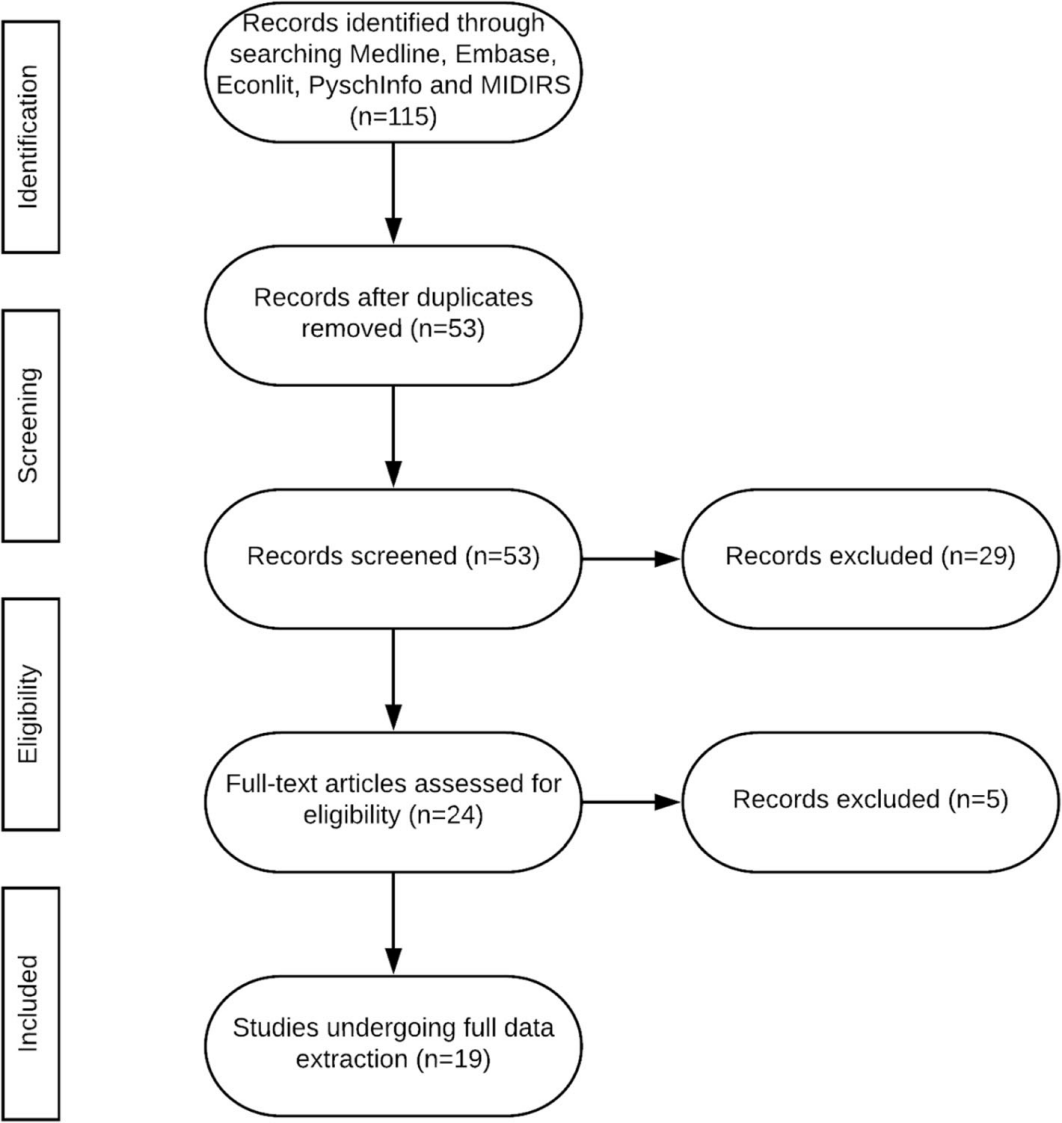
Flow of studies through the review

A total of 19 studies relevant to antenatal or newborn screening programmes were identified and underwent detailed data extraction. The flow of studies through the review are shown in Fig. 1. The review process also identified a study eliciting women’s preferences for prenatal tests in Iceland [33], published in Icelandic and therefore not meeting our inclusion criteria. A qualitative study identifying women’s preferences for prenatal testing was also identified, although it did not contain an empirical DCE, the authors stated the identified themes will be used as attributes and levels in a future study [34]. Furthermore, another study elicited parents’ preferences for research on newborn dried bloodspots although this study used a rating rather than discrete choice exercise [35].

### Study setting & objectives

The majority of included studies (*n* = 15, 79%) elicited preferences for antenatal screening or prenatal tests. Only four studies (21%) elicited preferences for newborn screening programmes [36–39]. Only one study used BWS [38] employing a profile case approach [40].

Most studies (*n* = 13;68%) were published in the last five years. The majority of DCEs were conducted in Europe (*n* = 12; 63%). One study was conducted in the US [38], one in Canada [36], one in Australia [41], one in Singapore [42] and one in China and Hong Kong [43]. Two studies (11%) compared preferences across multiple countries: Hill et al., (2015) who compared the preferences of people in Canada, Denmark, Iceland, Israel, Italy, the Netherlands, Portugal, Singapore, and UK; and Lewis et al., (2006) who compared preferences from the UK and Australia.

The average (mean) sample size was 584. Pregnant women (*n* = 11; 58%) and healthcare professionals (*n* = 11; 58%) were the most common samples. ‘Health care professionals’ referred to obstetricians, gynaecologists, nurses, midwives, sonographers or, sometimes, as simply ‘other’ with no clarification in the manuscript. Ten studies (56%) compared preferences across different samples [41, 42, 44–51]. Table 1 summaries key data extracted from the included studies.

**Table 1:** Summary of reported methods

Study (country, year)	Attributes	Sample	Design	Analysis
Barrett et al. [42] (Singapore, 2017)	Four: accuracy, time of results, risk of miscarriage, and amount of information provided.	Pregnant women (*n* = 69) and healthcare professionals (*n* = 301).	Ten unlabelled choices of two alternatives generated using an unreported design with level balance, minimal overlap and orthogonality and incorporating a test for internal validity.^a^	Conditional logit model
Beulen et al. [46] (the Netherlands, 2015)	Seven: minimal gestational age, time to test results, level of information, detection rate, false positive rate, miscarriage risk, and cost.	Pregnant women (*n* = 596) and healthcare professionals (*n* = 297).	Seventeen unlabelled choices of two alternatives and a dual non-response option generated using an unreported design with level balance, minimal overlap and orthogonality and incorporating a test for internal validity.	Conditional logit model
Bishop et al. [48] (UK, 2004)	Three: time of test, detection rate, and risk of miscarriage of a baby unaffected by Down’s Syndrome.	Pregnant women (*n* = 291) and healthcare professionals (*n* = 98).	Four unlabelled choices of two alternatives and a dual non-response option generated using an unreported design with unspecified methods and incorporating a test for internal validity.	Random effects probit model.
Boormans et al. [53] (the Netherlands, 2010)	Five: detection capacity, anxiety, waiting time, failure rate, and consequences of detected chromosomal abnormalities.	Pregnant women (*n* = 103).	Thirty-two labelled choices of three alternatives generated using a D-efficient design.	Conditional logit model.
Carroll et al. [52] (UK, 2013)	Four: detection rate, gestation, time to wait for results, and cost.	Women and partners (*n* = 103).	Sixteen unlabelled choices of two alternatives generated using a main effects design with maintaining orthogonality	Conditional logit and latent class models.
Chan et al. [43] (China, Hong Kong, 2009)	Three: level of the test information, waiting time for result availability, and cost of test.	Pregnant women (*n* = 300).	Eight labelled choices of three alternatives and an optout generated using a main effects design with level balance, minimal overlap and orthogonality and tested for non-traders always choosing a certain alternative or ‘no test.’	Conditional logit model with subgroup analysis.
Hill et al. [44] (Canada, Denmark, Iceland, Israel, Italy, the Netherlands, Portugal, Singapore, and UK, 2015)	Four: accuracy, time of test, risk of miscarriage, and provision of information about Down syndrome only, or Down syndrome and other conditions.	Pregnant women (*n* = 2666) and healthcare professionals (*n* = 1245).	Nine unlabelled choices of two alternatives and an optout generated using a main effects design with level balance, minimal overlap and orthogonality and incorporating a test for internal validity.	Conditional logit model.
Hill et al. [47] (UK, 2012)	Four: accuracy, time of test, risk of miscarriage, and provision of information about Down syndrome only, or Down syndrome and other conditions.	Pregnant women (*n* = 355) and healthcare professionals (*n* = 181).	Ten unlabelled choices of two alternatives generated using a main effects design with level balance, minimal overlap and orthogonality and incorporating a test for internal validity.	Conditional logit model.
Hill et al. [50] (UK, 2017)	Three: accuracy, time in pregnancy when the test result is received and risk of miscarriage.	Service users (carriers/affected with sickle-cell) (*n* = 67) and healthcare professionals (*n* = 62).	Eight unlabelled choices of two alternatives and an optout generated using a main effects design with level balance, minimal overlap and orthogonality and incorporating a test for internal validity. ^b^	Conditional logit model with subgroup analysis.
Hill et al. [49] (UK, 2014)	Three: risk of miscarriage, accuracy, and time in pregnancy when the test result is received.	Adult cystic fibrosis patients (*n* = 92), carriers (*n* = 50) and healthcare professionals (*n* = 70).	Eight unlabelled choices of two alternatives and an optout generated using a main effects design with level balance, minimal overlap and orthogonality and incorporating a test for internal validity.	Conditional logit model with subgroup analysis.
Lewis et al. [41] (Australia, 2006)	Three: risk of miscarriage, accuracy and time in pregnancy when the test result is received.	Pregnant women (*n* = 322), midwives (*n* = 266) and obstetricians (*n* = 34).	None unlabelled choices of two alternatives and a dual non-response option generated using an unreported design with maintaining orthogonality and incorporating a test for internal validity.	Random effects probit model.
Lewis et al. [45] (UK, Australia 2006)	Three: risk of miscarriage, accuracy and time in pregnancy when the test result is received.	Midwives (*n* = 146 in Australia, 53 in UK) And obstetricians (n = 29 from Australia, 41 in UK) .	Twelve unlabelled choices of two alternatives generated using an unreported design with unspecified methods and incorporating a test for internal validity.	Random effects probit model.
Lund et al. [51] (Denmark, 2018)	Four: accuracy, time of test, risk of miscarriage, and provision of information about Down syndrome only, or Down syndrome and other rare conditions.	Women (*n* = 315) and their partners (*n* = 102) in addition to foetal medicine experts and sonographers (*n* = 57) and midwives not involved in screening (*n* = 48).	Ten unlabelled choices of two alternatives generated using a main effects design with level balance, minimal overlap and orthogonality and incorporating a test for internal validity. ^c^	Conditional logit model with subgroup analysis.
Lynn et al. [54] (UK, 2015)	Four: health-care professional conducting the scan, detection rate for abnormal foetal growth, provision of non-medical information, and cost.	Pregnant women (n = 146)	Sixteen unlabelled choices of three alternatives and an optout generated using a main effects design with maintaining orthogonality and incorporating a test for internal validity.	Mixed logit model.
Miller et al. [36] (Canada, 2015)	Five: clinical benefits of improved health, earlier time to diagnosis, reproductive risk information, false-positive (FP) results, and overdiagnosed infants.	Members of the public (*n* = 1213).	Eight unlabelled choices of three alternatives and an optout generated using a D-efficient design.	Mixed logit and generalised multinomial logit models.
Ryan et al. [55] (UK, 2005)	Three: level of information, number of days’ wait for results, and cost to you.	Pregnant women (*n* = 40).	Eight labelled choices of two alternatives and an optout generated using an unreported design with unspecified methods.	Conditional logit model.
Tarini et al. [38] (USA, 2018)	Ten: number of babies diagnosed, chance of false positive, cost, likelihood of developing symptoms, seriousness of symptoms without treatments, age of symptoms and life expectancy without treatment, time to start of treatment, success of treatment, side effects of treatment, impact of diagnosis.	Members of the public (*n* = 502).	Four choice sets to select the ‘most important’ and ‘least important’ characteristic generated using an efficient experimental design.	Generalized estimating equation logit model.
Wright et al. [37] (UK, 2017)	Four: how information is provided, when information is provided, parents’ ability to make a decision, cost to the parents.	Current and future patients aged 18–45 (*n* = 702).	Ten unlabelled choices of three alternatives and an optout generated using a D-efficient design with Ngene and incorporating a test for internal validity.	Heteroskedastic conditional logit model.
Wright et al. [39] (UK, 2018)	Four: how information is provided, when information is provided, parents’ ability to make a decision, cost to the parents.	Midwives (*n* = 134).	Ten unlabelled choices of three alternatives and an optout generated using a D-efficient design with Ngene and incorporating a test for internal validity.	Conditional logit model.
^a^reported in [44]; ^b^reported in [49]; ^c^reported in [47].				

### Key findings

This review identified seven key aspects that were frequently included or discussed in the DCE or BWS exercises. These common attributes and a brief description of the key findings related to each are presented in Table 2.

**Table 2:** Summary of key findings

Investigation	Study findings
Accuracy of technology	Almost unanimously the most important factor for healthcare professionals. Also important to women but they will sacrifice accuracy for safety e.g. reduced risk of miscarriage.
When test/screening occurs	When the test occurs is a significant factor in women’s choices for screening. However, clinicians value this attribute much more. Some authors hypothesise this is because women are uninformed about the consequences of late testing (for treatment/termination choices).
Level and/or type of information	This is very mixed, with some studies finding more information to be of negligible/no value and others finding it highly valued. The studies which considered this attribute were sometimes unlabelled (test A etc) or sometimes labelled (karyotyping, rapid aneuploidy detection) so women may think more information means more invasive or more painful screening procedures?
Time to results	This is generally important in women’s decision to participate in screening however it is generally of low value. For a very small proportion of the population, this has been found to be the most important factor.
Cost	Only included in a few studies but is highly important to a large price sensitive part of the population.
Risk of harm	Almost unanimously the most important factor to women. Almost all studies find this is highly valued compared to other attributes.
Preference heterogeneity	Some studies have found heterogeneity between healthcare professionals and women whereas others have found preferences to be homogeneous. Differences in preferences may not exist due to the analyses conducted by authors.

#### Accuracy of technology

The most commonly occurring attribute was accuracy (*n* = 15; 79%). However, only three of these studies explained this in detail; Tarini et al. [38] and Beulen et al. [46] explained this in terms of the chance of a false positive and Carroll et al. [52] defined accuracy by a true positive rate.

Two studies, [42, 51]**,** concluded accuracy of the test was the most important attribute for healthcare professionals (whereas women sampled favoured ‘safety’). Similarly [46] found healthcare professionals were willing to pay €138 for a 1% increase in detection and €267 for a 1% lower false positive rate whereas women were willing to pay €53 and €112, respectively. However, Boormans et al. [53] which only considered women’s preferences, found the test’s detection capacity to be one of the most important attributes particularly compared to the less valued waiting time and anxiety. Another study [52] also found that in one of their preference classes (accounting for 43% of the sample), detection rate was the most important attribute.

#### When screening/testing occurs

When the screening occurred was included as an attribute in 13 studies (68%). Time of results was included as an attribute in [42] but was not statistically significant in most of their analyses. Carroll et al. [52] found that although an earlier gestation was preferred in most cases, it was not a strong preference in any of their analyses. Similarly, Beulen et al. [46], found time of testing was statistically significant but women were only prepared to pay €23 for testing 1 week earlier in pregnancy (compared with €905 to reduce the risk of a miscarriage by 1% and €1200–€1400 for information on trisomies 13, 18, 21 and other abnormalities of DNA). Another study [48] also found women placed less value on early tests compared to risk of miscarriage and detection rate, and less value in comparison to healthcare professionals.

#### Type of information

Ten studies (53%) had an attribute regarding the level or type of information provided [36, 37, 39, 42–44, 46, 47, 51, 54, 55]. Although level of information (trisomies 21, 18 and 13 only, or these three aneuploidies plus additional information on other chromosomal abnormalities) was included by [42], the attribute was not statistically significant in most of the analyses. In contrast, another study [46] found additional information to be the highest valued attribute. With women prepared to pay an additional €1200–€1400 for information on trisomies 13, 18, 21 and other abnormalities of DNA changes over trisomy 21 only. Lund et al. [51] conducted subgroup analyses and found women who had undergone fertility treatment, experienced results which suggested they were at high risk, or those who had invasive testing placed a higher weight on comprehensive genetic information than women who conceived naturally or had no experiences of test risks.

#### Time to results

Time to results appeared as an attribute in six (32%) studies [43, 46, 48, 52, 53, 55], although Tarini et al. [38] included the attribute ‘time to start of treatment’. Beulen et al. [46] found waiting time to be statistically signficant but it was the lowest valued attibute for the pregnant women and clinicians in their sample. Similarly, [53] also found that waiting time was much less valued compared to other attributes such as detection or consequences of a chromosal abnormality.

#### Procedure-related risks

Risk of miscarriage was presented as an attribute in ten (53%) studies [41, 42, 44–51]. Barrett et al. [42], Lund et al. [51] and Beulen et al. [46] found that the risk of a miscarriage was the most important factor in women’s choices, whereas healthcare professionals prioritised test accuracy. Similarly, Bishop [48] found women would wait twice as long for the test as healthcare professionals (4 v 2 weeks) for a 1% reduction in the risk of a miscarriage.

#### Cost of participation

Cost was included as an attribute in seven (37%) studies [37–39, 43, 52, 54, 55] to the person consuming the test (parent(s)) even when the respondents were healthcare professionals [39, 46]. Carroll et al. [52] found cost to be the most important attribute, but after investigations into heterogenetiy concluded that this was driven by one large preference group.

### Overview of methods

The average (mean) number of choice-sets respondents were asked to complete was eleven. Although this ranged from four [38, 53] to 32 [48]. Only three studies (16%) used a labelled design [43, 53, 55] where the type of test (e.g karyotyping) described the alternative. Three (16%) studies [41, 46, 48] offered a ‘dual non-response’ so respondents could express they felt indifferent and/or would not choose any of the alternatives presented. Nine (47%) studies offered respondents the option to opt-out [36, 37, 39, 43, 44, 49–51, 54, 55].

Details about the experimental designs were generally sparsely reported in the studies included in this review. Four (21%) studies [36, 37, 39, 53] used D-efficient experimental designs and eight (42%) studies [42–44, 46, 47, 49–51] ensured level balance, minimal overlap and orthogonality.

The most popular (*n* = 11; 58%) analytical model was a standard conditional logit model, with one study [37] extending this to allow for heteroscedasticity in the error term. The single BWS study analysed the choice data using generalized estimating equation logit model. Five studies (26%) used random parameter models [36, 41, 45, 48, 54] and one study [43] used latent class analysis to explain the variation in preferences.

Only two studies [36, 37] employed models which allowed for the variance of the error term to differ across individuals. Other studies also pooled data from different sources, merging data from ‘healthcare professionals’ whether these were midwives, nurses, obstetricians or ‘other’ or directly comparing coefficients across subgroups. As a result, some studies [41–45, 47–49, 51] may also have erroneously concluded that there were differences in preferences. In some articles, the authors [42, 50] acknowledged in the methods section marginal rates of substitution ‘allowed for comparison of different attributes using a common scale’. When reporting marginal rates of substitution calculations, a number of studies [41, 44, 45, 47–50, 54] simply presented this as ratio of the two coefficients which was assumed to be statistically significant with no confidence intervals for the ratio reported.

There was generally little detail provided on the alternative specific constant (ASC). This term is used to capture the mean of the error term and therefore describes the utility not described by the attributes included in the choice experiment. In the case of an opt-out alternative, the ASC may represent the utility (if positive) of opting out or the ‘disutility’ of missing out on the good/service being offered (if negative). In some studies there was no detail [44, 47, 49], and in others [41, 45, 48] it was presented in the footnote of a table with no indication of its statistical relevance (significance, standard errors *etc*). In another study, [54] the constant term was not specified in the estimated utility function (equation one) but was reported as a coefficient in the results table.

## Discussion

This review identified 19 studies that aimed to quantify preferences for aspects of antenatal and newborn screening. Accuracy of the test or screening programme was the most commonly included attribute. In contrast to advice in the risk communication literature, but in line with other healthcare DCEs [56], risk attributes (including risk of miscarriage) were most commonly presented as a percentage. In only three studies was ‘accuracy’ broken down into sensitivity or specificity. There is some evidence that individuals have different preferences for sensitivity and specificity and the balance of these aspects of antenatal and newborn screening require further investigation [57]. In addition, individuals find risk and percentages complex attributes to understand [58]. Studies comparing preferences frequently concluded that accuracy was relatively more important to healthcare professionals than women. However, it is unclear whether this is because of differences in each sample’s interpretation of this information. Future research could investigate if the heterogeneity is robust to different formats or more detailed explanations of the accuracy information.

Understanding if, and how, preferences for screening are affected by information and the communication of probabilities may have implications beyond completing a valuation study such as a DCE; for example, the preparation of invitations and screening leaflets or tailoring these to target subgroups. Several studies have explored how to facilitate informed decision making in antenatal and newborn screening [59, 60]; however, there appears to be no “one size fits all” approach [61]. It has been suggested that risk information should be tailored but there is a great challenge in adapting to the social, religious and cultural background of the user(s) or their attitude or knowledge [15]. In this review, Beulen et al. [46] and Barrett et al. [42] both suggested personalised counselling for women when providing information about or results from prenatal testing.

Other commonly occurring attributes were when the screening or testing occurred and time to results. Studies which included these attributes often found that they were not statistically significant or relatively unimportant in women’s decision-making. This could be because the advantages of early diagnosis and treatment may have been unclear, uncertain or unknown. Carroll et al. [52] found that in one of their preference classes waiting time was the most important attribute, however, this group accounted for less than 12% of the sample. For prenatal screening, the importance of early testing or rapid results for decisions about pregnancy termination may be irrelevant to some groups of women; future studies should consider heterogeneity in preferences around these aspects of screening.

Commissioners of screening programmes need to determine which screening programmes should be provided and by whom, when the tests should be offered, and how information should be given and consent obtained. No studies included in this review predicted demand or uptake for services or screening programmes. The validity of the included studies was also not explicitly discussed, despite uptake rates of screening being published in many countries [62, 63] and a desire to understand if stated preferences match those ‘revealed’ by the individual in the real-world [64]. Uptake rates can also be used for model parameterisation [65] particularly for new technologies where demand may be unknown. Similarly, only six studies included a cost attribute to calculate willingness-to-pay. Monetary valuation of aspects of screening or the programmes as a whole can be useful for comparing across subgroups but also in cost-benefit analysis to understand, for example, the net-benefit of expanded programmes [66].

In this review, only two studies specifically considered partners’ views [51, 52], although some studies with a broader sampling strategy of the public may have picked up these opinions indirectly [36–38]. Partner preferences may be an important consideration when understanding choices about screening, and there is evidence to suggest partners may have different decision-making processes with different reactions to test information [67]. Future studies may wish to consider the preferences of partners and investigate if, and how, they are related to the mother’s choices.

Researchers seeking to contribute to the development of evidence reporting preferences for antenatal and newborn screening services should make use of advancements in experimental designs, for example using Bayesian or D-efficient approaches [68], to ensure attribute or level interactions of interest can be measured. Although the experimental design often included the option of ‘no screening’, the coefficient on the constant term was rarely reported in the results tables. This provides important information about women’s preferences in general for/again screening and includes information about the role of other attributes not included in the choice set. The studies included in the review also had large samples when compared with standard healthcare DCEs so there was potential to conduct sophisticated analyses and investigate preference heterogeneity either through subgroup analysis or other model specifications. Although not always considered in the studies included in this review, understanding the degree of heterogeneity in individuals’ preferences for healthcare could help decision-makers configure screening services to improve uptake and the utility of those who have participated.

The largest methodological issues related to 1) scale heterogeneity and 2) calculation of marginal rates of substitution. The pooling of data from multiple sources and assumption that the error term is homoscedastic, meant that the coefficients of the pooled models were uninterpretable. Methods which allow researchers to disentangle issues of scale include the heteroskedastic conditional logit or [69] generalised multinomial [70] models or scale-adjusted latent class analysis [71]. Alternatively, researchers can compare ratios of coefficients in marginal rates of substitution such as willingness-to-pay or willingness-to-wait. In this review, some studies reported point estimates of these ratios, presumably assuming the ratio of two significant coefficients could be interpreted as significant too. The Delta or Krinsky Robb methods [72] can be used to estimate the confidence intervals for a ratio, revealing to readers the degree of uncertainty in the trade-off calculations [73].

### Limitations

The review focussed on choice experiments (DCE and BWS) and did not include other types of preference elicitation methods such as contingent valuation, time trade-off or standard gamble studies. The focus of this review on attribute-based choice experiments allowed synthesis of the evidence in terms of the key aspects of screening which would have been challenging with contingent valuation studies. Similarly, time trade-off and standard gamble approaches are more commonly used for health state valuation. The review focussed on published materials and grey literature was not included; another possible limitation. The most serious limitation of this review was the reliance on study reports. Generally, there were little details on the methodological component of the study with quantitative tests (such as for scale heterogeneity) unreported. Therefore the findings presented in Table 2 may not be correct if the authors of the included studies have drawn erroneous conclusions from their data.

## Conclusion

This review has shown that DCEs and BWS are currently being under used to understand preferences for antenatal and newborn screening programmes. Multiple studies concluded that accuracy was the most important aspect of testing to healthcare professionals whereas women placed more importance on the risks of participation. Further research is required to understand if these valuations are robust to different approaches to framing information. Limited reporting of the methodological component in some studies made interpretation of the findings challenging and in future studies, more sophisticated approaches to experimental design and/or the discrete choice modelling may improve confidence in the results. Researchers wanting to use DCEs for future applications in this area may want to compare estimated demand to actual participation rates as a test for study validity and generalisability. Furthermore, these estimates could be used by decision-makers to configure screening services to maximise uptake and, as a consequence, the health benefit to the population.

## Abbreviations

BWS: Best-worst scaling
DCE: Discrete choice experiment
DNA: Deoxyribonucleic acid
NBSP: Newborn bloodspot screening programme
NIPT: Non-invasive prenatal testing
PKU: Phenylketonuria
WHO: World Health Organisation
